# Feasibility Analysis of Calcium Carbonate Particle Trajectory Simulation in a Dual Horizontal Shaft Mixer

**DOI:** 10.3390/ma16175999

**Published:** 2023-08-31

**Authors:** Guozheng Song, Faguo Huang, Jiafang Pan

**Affiliations:** 1 Key Laboratory of Advanced Manufacturing and Automation Technology, Education Department of Guangxi Zhuang Autonomous Region, Guilin University of Technology, Guilin 541006, China; sgz@glut.edu.cn (G.S.); huangfaguo@glut.edu.cn (F.H.); 2 Guangxi Engineering Research Center of Intelligent Rubber Equipment, Guilin University of Technology, Guilin 541006, China

**Keywords:** discrete element software, mixing uniformity, EDEM, trajectory simulation, simulation feasibility analysis

## Abstract

This article aims to investigate the feasibility of using discrete element software EDEM 2022.0 to simulate the trajectory of artificial marble patterns in a dual horizontal shaft mixer. Research was conducted on the mixing uniformity of particles in the mixing chamber, and the optimal speed range for particle mixing was established. By simulating the trajectory of pigment particles, the trajectories of the particles at different positions of the stirring paddle were obtained, and the trajectories were compared with the measured results. In the study of uniform particle mixing, the Lacey index at different speeds was compared, and the optimal speed range was established between 40 RPM and 60 RPM. Based on this, the particle trajectory simulation found that the motion trajectories of particles at different positions of the stirring paddle varied significantly. The particles in the stirring paddle rod exhibit a gradual trend, in which they gradually decrease as they approach the head of the stirring paddle. Finally, the feasibility of this method was established by comparing the simulated and actual patterns through proportional replication of the mixing process, and it was discovered that the two were similar.

## 1. Introduction

Imitation marble is made of stone powder and stone sand after the crushing of natural calcium carbonate rock (hereinafter referred to as the base material), through the use of unsaturated polyester resin transfer molding and vacuum vibration compression technology to obtain high hardness, high temperature resistance, high corrosion resistance, and other advantages, and is widely used in the construction and decorative products industry [1,2]. In addition to retaining the natural color of the rough stone, imitation marble can also be added with pigments of different colors to enrich the diversity of its color during the production process and form a variety of pattern textures to obtain a more competitive appearance and beauty than natural stone.

The pattern is formed by mixing and stirring the pigment with the marble based on uniform mixing of the silt substrate, and the distribution state of the stirring trajectory of the pigment in the substrate forms the pattern texture of imitation marble. This process is called the stirring molding of patterns. Mixing is the process by which a system evolves under agitation from one simple state (where the components are initially separated) to another simple state (where the components are perfectly aligned) [3]. Mixing quality plays a vital role in the production process [4,5], the position of the particles will continue to change during the mixing process when different kinds of particles are in the same volumes with the same proportion or close. Its state is uniform mixing, and the mixing state of the material determines the quality of imitation marble production and processing. In this paper, the mixing of particles is investigated using the EDEM software [6]. In the manufacturing process of imitation marble, the twin-shaft mixer is the key process equipment, which not only affects the product quality but also plays a decisive role in the mixing and molding of the pattern. The core mechanism of pattern stirring molding lies in the stirring movement mechanism of particles. Due to the lack of theoretical research on imitation marble pattern stirring molding at this stage, the pattern molding process in the industry still stays at a low level of relying on manual experience, resulting in the instability and irreproducibility of the pattern molding process, which brings great obstacles to the development of the custom molding process technology of the product.

To solve this problem, this paper assesses the feasibility of imitation marble pattern simulation analysis and molding mechanism. Firstly, the optimal speed range is established by analyzing the influence of the speed of the stirring paddle on the mixing uniformity of particles, and the prerequisites for the analysis of particle trajectory are provided. Secondly, through the simulation analysis based on the discrete element method, the pattern-forming process of the “Aimeiyu” product is simulated and the formed pattern is compared with the pattern of the actual product to explore the feasibility of using the simulation software EDEM to simulate the pattern of imitation marble. Then, the trajectory of single particles on the paddle head, shaft, paddle root, and mixing shaft is simulated, and the trajectory change trend of the particle motion at different positions is obtained. Finally, by comparing with the production process of imitation marble, the movement trajectory change law of particles at different positions of the mixing paddle is verified to provide a theoretical reference for the customized molding of the pattern.

## 2. Related Theories

### 2.1. Discrete Element Method

The discrete element method (DEM) is a numerical simulation method used to analyze the dynamics and mechanical properties of complex particle systems and has been widely used in the analysis of motion between particles and particles and between particles and machinery [7,8,9,10,11,12,13,14,15]. TING J et al. [16] introduce in detail the development of discrete element two-dimensional elliptic particle numerical algorithms, and the changes between particles and particles and between particles, velocity, and force; “Equations that determine particle-to-particle, particle-to-wall contact position, and velocity increments are derived and integrated into traditional DEM algorithms.” The results show that the mechanical behavior obtained by applying the described method is more quantitatively and qualitatively similar to the behavior of real materials compared with the use of round particles. Fenglei Qi et al. [17] used the DEM to simulate particle flow and studied the mixing performance of a laboratory-scale twin screw mixer by using glass beads instead of particles. The results show that compared with the experimental research, DEM provides more information about the particle flow in the mixer, such as particle trajectory, particle velocity, and local mixing state, which are either economical and expensive or cannot be measured with existing experimental techniques. Coetzee, CJ [14], through a review of the literature over the past 25 years, discussed the calibration of specific parameters, and the results are of great help to future researchers in improving calibration methods and laying a parameter foundation for better use of DEM methods. Muhammad Kashif Saeed et al. [18] used the DEM method and the open-source simulation software LIGGHTS 3.8.0 to study the flow and mixing behavior of polydisperse non-spherical non-viscous particles in cylindrical trough mixers. As a result, in practice, non-spherical particles exert more forces and stresses in the system than spherical particles. Mohammadreza Ebrahimi et al. [19] used sampling experimental techniques and discrete element methods to study the mixing of bidis perse particles in a horizontal paddle mixer. A series of experimental data were used to calibrate and verify the DEM model, and the numerical simulation results were in good agreement with the experimental measurement results, which verified the reliability of the numerical simulation.

### 2.2. Particle Contact Model

By using the discrete element method, the position information of particles can be tracked, and the movement of particles obeys Newton’s second law. The choice of contact model determines the interaction relationship between particles.

Since the powder particles themselves are not vicious, the addition of resins and catalysts makes the particles viscous. Rajulu, A.V. et al. [20] demonstrated that the co-blended epoxy-unsaturated polyester resins were fully miscible using viscosity, refraction, etc. Soo-Jin Park et al. [21] concluded that the presence of 5% unsaturated polyester in the epoxy resins was effective in obtaining the optimum toughness of the castings. Soo-Jin Park et al. [22] experimentally verified that the addition of 5% unsaturated polyester to epoxy resins with the addition of 5% epoxy resin resulted in improved tensile strength, flexural strength, compressive strength, impact strength, and water absorption properties, compared to coupling agent treated granite powder epoxy composites. It was also found that the bonding of 5% unsaturated polyester toughened epoxy granite powder composites with the matrix was superior to that of other composites, and the addition of inexpensive fillers had the advantage of low overall cost. Therefore, the resin used in the artificial calcium carbonate industry is mostly a blended solution of unsaturated polyester resin and epoxy resin. The viscosity and self-solubility of the blended solution forces the particles to bond with each other.

To quantify the viscosity in the contact state, JKR (Hertz-Mindlin with JKR) contact theory is used for DEM simulation [23,24,25,26]. The JKR model has been widely used in a variety of DEM models that consider cohesion [27,28,29,30,31,32,33], hence the JKR model is chosen as the contact model for particle mixing motion in the mixer.

**Figure 1:**
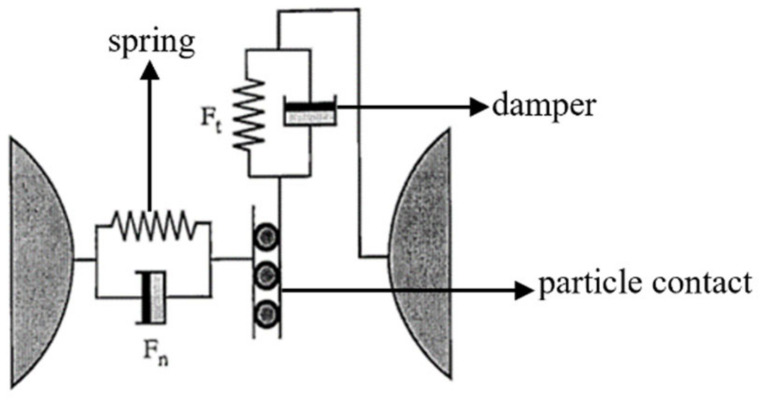
Particle contact mechanics model.

The modeling technique uses the soft particle method [8], which allows overlapping between particles. The collision force F is determined by the normal and tangential forces F_t_, and the particle contact mechanics model is shown in Figure 1. The normal force and the tangential force Ft provide the repulsive force through the spring and the damper provides the dissipation, resulting in an effective recovery coefficient.

In DEM, the control equations for a single particle can be written as [34]:

(1) $$m_{i}\frac{d\vec{v_{i}}}{dt}=\underset{j\neq i}{\overset{k}{\Sigma }}{\vec{F}}_{ij}+m_{i}\vec{g}$$

(2) $$I_{i}\frac{d{\vec{\omega }}_{i}}{dt}=\underset{j\neq i}{\overset{k}{\Sigma }}{\vec{T}}_{ij}$$

(3) $${\vec{F}}_{ij}={\vec{F}}_{ij}^{n}+{\vec{F}}_{ij}^{t}$$

(4) $${\vec{T}}_{ij}={\vec{R}}_{i}\times {\vec{F}}_{ij}^{t}-{\vec{\tau }}_{ij}^{r}$$

(5) $${\vec{F}}_{ij}^{n}=-K_{n}\delta_{n}n_{c}-C_{n}\left(v_{c}\cdot n_{c}\right)n_{c}$$

(6) $${\vec{F}}_{ij}^{t}=-K^{t}v_{c}^{t}+C_{t}\left(v_{c}\times n_{c}\right)\times n_{c}$$

where $m_{i}$, ${\vec{v}}_{i}$, ${\vec{\omega }}_{i}$, ${\vec{R}}_{i}$, and $I_{i}$ are the mass of the particle i, the translational speed, the rotational speed, the vector of the center of the particle i and the contact point, and the moment of inertia of the particle i. The contact force caused by particle j can be divided into two parts: the normal contact force and the tangential contact force. ${\vec{T}}_{ij}$ is the torque of the particle j due to tangential contact force and rolling friction. The total contact force and the total torque are the sum of k particles in contact with i particles.

$K_{n}$ is the normal spring coefficient, $\delta_{n}$ is the normal displacement, $C_{n}$ is the normal damping coefficient, $v_{c}$ is the relative velocity, and $n_{c}$ is the unit normal vector. $K^{t}$ is the tangential spring coefficient, $v_{c}^{t}$ is the tangential velocity, and $C^{t}$ is the tangential damping coefficient. The left hand side of Equation (5) represents the spring force, which is proportional to the normal displacement; the right hand side of the equation represents the damping force, which is proportional to the normal component of the relative velocity. The left hand side of Equation (6) represents the spring force, which is inversely proportional to the tangential velocity; the right hand side of the equation represents the damping force, which is proportional to the cross product of the relative velocity and normal vector, and then to the cross product of a normal vector.

In the JKR model, the contact force generated by the contact between viscous particles can be divided into two types: normal and tangential. The normal contact force consists of the normal elastic contact force and the normal damping force, and the tangential contact force consists of the tangential elastic force and the tangential damping force, and the expressions are as follows:

(7) $$F_{co}=F_{con}+F_{\cot}$$

(8) $$F_{con}=F_{ecn}+F_{dn}$$

(9) $$F_{\cot}=F_{et}+F_{dt}$$

Formula: $F_{co}$ represents the contact force; $F_{con}$ and $F_{cot}$ represents the normal contact force and the tangential contact force, respectively; $F_{ecn}$ and $F_{dn}$ are the normal elastic contact force and normal damping force; and $F_{et}$ and $F_{dt}$ represent the tangential elastic force and tangential damping force.

In the JKR model, the normal contact force is related to the contact radius and surface energy (cohesion energy) due to the appearance of viscous forces between particles.

(10) $$F_{ecn}=-4\sqrt{\pi \gamma E^{*}}a^{3/2}+\frac{4E^{*}}{3R^{*}}a^{3}$$

(11) $$\sigma =-\sqrt{\frac{4\pi \gamma a}{E^{*}}}+\frac{a^{2}}{R^{*}}$$

$F_{ecn}$ represents the normal elastic contact force, $R^{*}$ is the equivalent radius of the particle, σ is the degree of overlap between the spheres in the contact, γ is the surface capacity, and a is the contact radius. The equivalent Young’s modulus $E^{*}$ is as follows:

(12) $$\frac{1}{E^{*}}=\frac{1-\nu_{i}^{2}}{E_{i}}+\frac{1-\nu_{j}^{2}}{E_{j}}$$

(13) $$\frac{1}{R^{*}}=\frac{1}{R_{i}}+\frac{1}{R_{j}}$$

*E* is Young’s modulus, *ν* is Poisson’s ratio, and the subscripts *i* and *j* represent the two particles in contact with each other, respectively.

(14) $$a_{0}={\left\{\frac{9\pi \sigma {\left(R^{*}\right)}^{2}}{E^{*}}\right\}}^{1/3}$$

$a_{0}$ is the contact radius at equilibrium. The amount of overlap is related to the contact radius and the contact radius at equilibrium as follows:

(15) $$\delta_{n}=\frac{a^{2}}{R^{*}}\left\{1-\frac{2}{3}{\left(\frac{a}{a_{0}}\right)}^{-3/2}\right\}$$

*a* is the normal overlap. The normal damping force depends on the velocity of the particles and is calculated as follows:

(16) $$F_{dn}=-2\sqrt{\frac{5}{6}}\frac{\ln\left(e\right)}{\sqrt{\ln^{2}\left(e\right)+\pi^{2}}}\sqrt{S_{n}m^{*}}v_{n}$$

(17) $$S_{n}=2E^{*}\sqrt{R^{*}\delta_{n}}$$

$F_{dn}$ is the normal damping force, *e* is the coefficient of recovery, $v_{n}$ is the relative velocity normal component of the particle, and $S_{n}$ is the normal stiffness.

(18) $$F_{et}=8E^{*}\sqrt{R^{*}\delta_{n}}\delta_{t}$$

(19) $$F_{dt}=-2\sqrt{\frac{5}{6}}\sqrt{S_{t}m}v_{t}$$

(20) $$S_{t}=8G^{*}\sqrt{R^{*}\delta_{n}}$$

where $F_{et}$ is the tangential elastic force, $F_{dt}$ is the tangential damping force, $S_{t}$ is the shear stiffness in the tangential direction, $G^{*}$ is the equivalent shear modulus, $\delta_{t}$ is the tangential overlap, and $v_{t}$ is the tangential relative velocity of particles.

### 2.3. Quantitative Analysis Method of Particle Mixing Uniformity

Quantitative analysis of the mixing uniformity of particles is an effective means to visually judge the mixing degree between particles. The degree of particle mixing is generally determined by the standard deviation method, coefficient of variation method, contact number method, Lacey index evaluation method, etc.

(1) The standard deviation evaluation method is to obtain the standard deviation by obtaining multiple sets of sample data and to describe the degree of inhomogeneity between particles in the particle system, and its expression is as follows:

(21) $$s=\frac{\sqrt{{\left(x_{1}-\bar{x}\right)}^{2}+\left(x_{2}-\bar{x}\right)+\cdots +{\left(x_{n}-\bar{x}\right)}^{2}}}{n-1}$$

(22) $$\bar{x}=\frac{x_{1}+x_{2}+x_{3}+\cdots +x_{n}}{n}$$

where *s* represents the standard deviation. $x_{1}$, $x_{2}$, etc. represent the sample data, and $\bar{x}$ represents the sample mean.

(2) The coefficient of variation method ($C_{v}$) is also known as the relative standard deviation, and its expression is as follows:

(23) $$C_{v}=\frac{s}{p}\times 100\%$$

where *s* is the standard deviation and *p* is the scale parameter.

(3) The contact number method [35] is a method of evaluating the degree of mixing based on the number of contacts, and its expression is:

(24) $$q=\frac{C_{sl}}{C_{total}}$$

where *C_sl_* is the number of contacts between different particles and *C_total_* is the number of contacts of the total particles.

(4) The Lacey index evaluation method is a hybrid method proposed by Lacey [36] through the use of statistical theory. The Lacey index is defined as follows:

(25) $$M=\frac{VAR_{0}-VAR}{VAR_{0}-VAR_{R}}$$

where *VAR* is the actual variance of the mixture; *VAR*_0_ is the variance in a completely separate system; and *VAR_R_* is the variance in a fully mixed system.

The standard deviation evaluation method is a method to evaluate the degree of mixing between non-homogeneous particles by the standard deviation of multiple sets of sample data. This method has the advantages of ease of operation and easy to understand, but its accuracy is related to factors such as the size and number of particles in the sample, and it ignores the size of the measured value itself. When studying non-homogeneous particles with large differences in component content in the mixed materials, the standard deviation is difficult to fully represent the mixing uniformity of the particle system. To compensate for this problem, the coefficient of variation method further considers the influence of the average value on the mixing uniformity based on the standard deviation, but it is more suitable for describing the axial mixing degree of particles in the drum. The contact number rule represents the mixing uniformity of the entire particle system with the number of contacts between non-homogeneous particles, but it is difficult to obtain the number of collisions between different particles in the experiment.

The Lacey index evaluation method combines various factors such as particulate matter concentration and particle size distribution, and it adopts scientific calculation methods to comprehensively consider various factors and standardizes the evaluation results to avoid the interference of subjective factors. The Lacey index evaluation method is suitable for the evaluation of different types of particulate matter mixed environments. The data acquisition is relatively easy, the calculation formula is simple, and it is easy to operate and understand. Therefore, the Lacey index is selected as the criterion for determining mixing uniformity.

## 3. Model Establishment of DEM Simulation 

### 3.1. Mixing Silo

**Figure 2:**
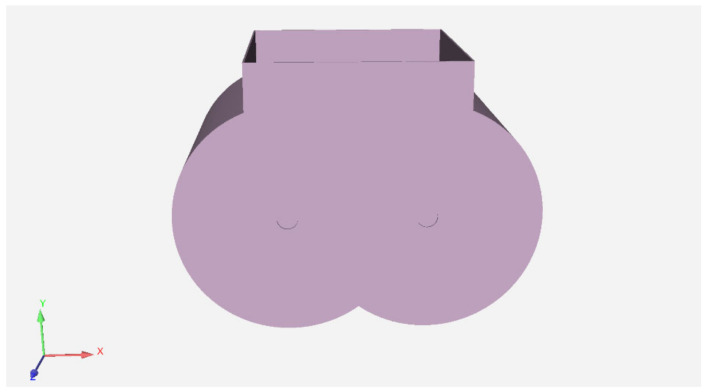
Simulation model of the mixing chamber.

The actual mixing chamber geometry is reduced to a three-dimensional model as shown in Figure 2.

The parameters of the mixing bin are shown in Table 1 [37,38].

### 3.2. Mixing Paddle

**Figure 3:**
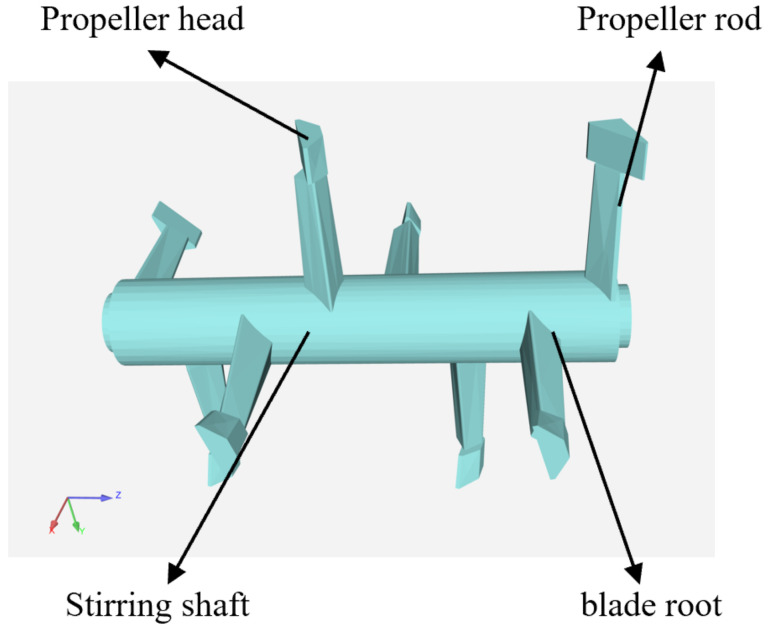
Structure of the mixing paddle.

The structure of the mixing paddle affects the movement trajectory of particles in the mixing chamber. In the simulation experiment, the mixing paddle is simplified to the three-dimensional model shown in Figure 3, and the simulation parameter settings of the mixing paddle are consistent with those of the mixing chamber. From the mixing paddle model, it can be seen that there is a declination angle between the paddle head and the paddle shaft, the shaft structure is diamond-shaped, the volume gradually decreases from the paddle root to the paddle head connection end, and the paddle root is connected to the mixing shaft.

### 3.3. Granular DEM Model

This article uses a three-ball and four-ball composite spherical particle model to analyze particle mixing uniformity and particle trajectory. Due to the complex shape and small particle size of marble after crushing and grinding along with the addition of unsaturated resin in production and processing, the substrate particles become clumpy. For the convenience of simulation analysis, a three-ball and four-ball composite spherical particle model is used to replace this shape. The particle parameters are shown in Table 2 [39].

The materials represented by the two composite particle models in each experiment are shown in Table 3.

## 4. Feasibility Study of Simulation Experiment

### 4.1. Feasibility of Substrate Blanking Filling Simulation Experiment

**Figure 4:**
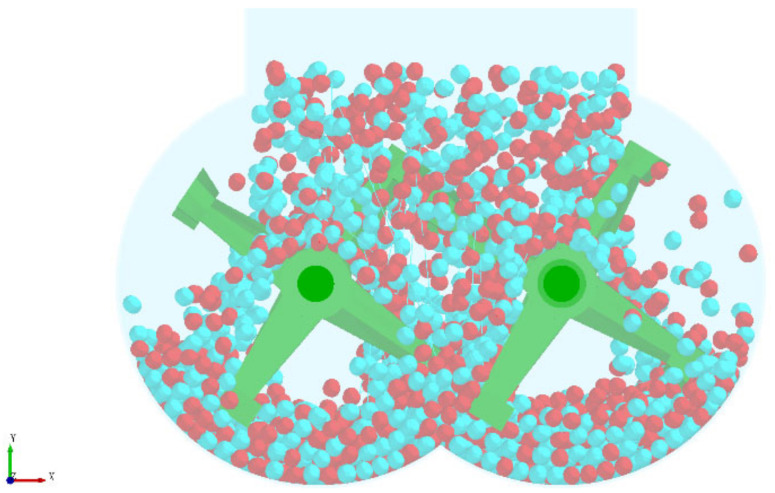
Blanking process of simulation experiment.

**Figure 5:**
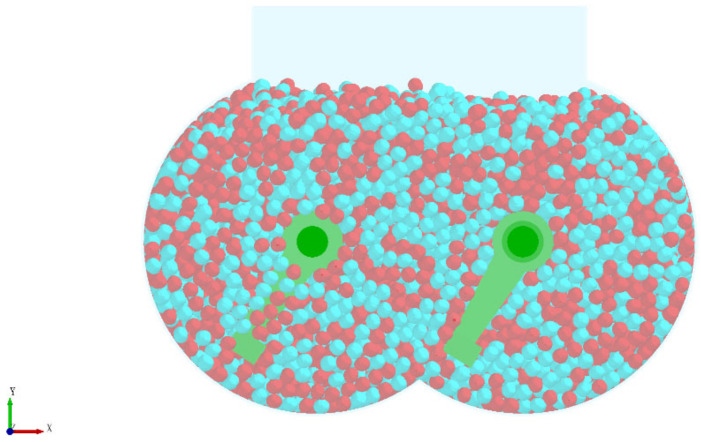
Filling effect after cutting.

In the actual production, the substrate is conveyed from the silo to the mixing silo through a belt conveyor. Due to the height difference between the feed inlet and the bottom of the mixing chamber and the light mass of the substrate particles, the trajectory deviation occurs during the falling of the substrate, resulting in the first disorderly mixing. The particle random generation module in the discrete element software is selected to simulate the initial mixing process to make the simulation experiment closer to the actual production process. To facilitate observation, the two particles were colored separately, and the unloading process of the simulation experiment is shown in Figure 4 and the substrate particle filling effect after the unloading is shown in Figure 5.

**Figure 6:**
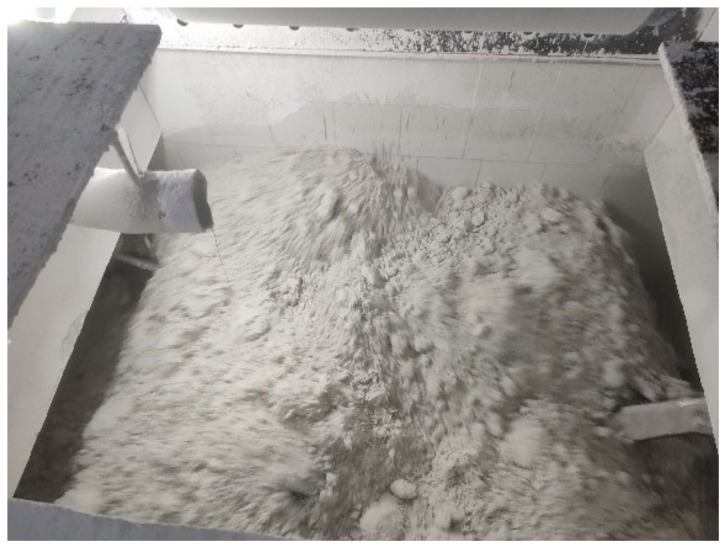
Diagram of the base material filling in the actual production.

In the actual production, the filling effect of the substrate is shown in Figure 6.

By comparing Figure 5 with Figure 6, it can be found that the simulation experiment of substrate particle filling is close to the filling effect in the actual production, and the results show that it is feasible to simulate substrate blanking filling through this simulation experiment.

### 4.2. Mixing Uniformity Analysis

The homogeneous mixing of the substrate is a prerequisite for model mixing and molding, hence the stirred particles are divided into several grids and part of the meshes are regularly selected as samples for evaluation. Due to the large particle size in the experiment, the dense meshing can easily lead to too few particles in the grid, hence the grid is divided into 5 × 7 × 5 sizes. To ensure that the selected grids are representative, odd numbers are taken in the X, Y, and Z directions for a total of 36 meshes.

**Figure 7:**
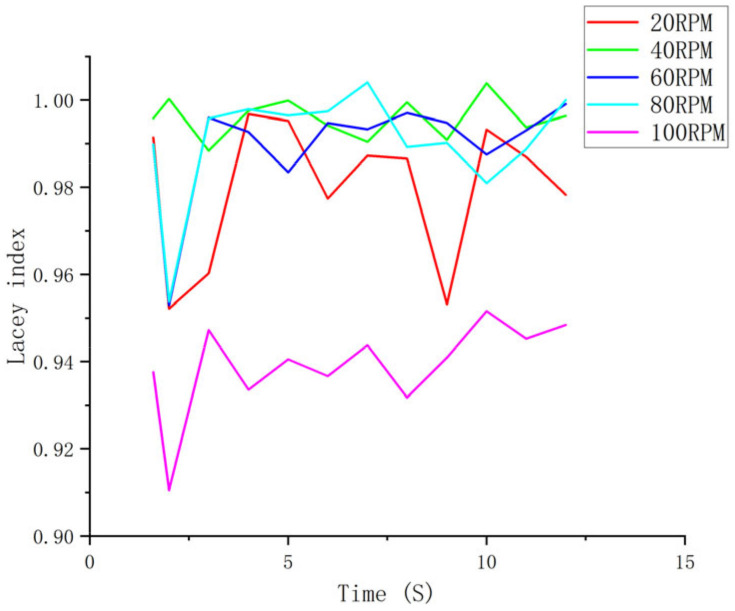
Lacey curves at different impeller speeds.

The Lacey index changes with the stirring time and is more pronounced in the segmented trend shown in Figure 7. This is because the simulation process converts the actual silt into a particle cluster, making it difficult to reflect the mixing state between small particles and it can only reflect the general mixing trend, which results in high and fluctuating values in the initial stage of the Lacey index. With the increase of time, the trough value gradually increases, indicating that the overall mixing uniformity is gradually increasing.

The simulation results show that the mixing uniformity of the particles at different speeds increases with time as shown in Figure 7, in which the mixing uniformity changes with time at 40 rpm and 60 rpm. The mixing is more stable, and the mixing effect decreases when the speed exceeds 60 rpm. When the speed reaches 100 rpm, although the mixing uniformity increases with the increase in stirring time, the mixing effect is the worst at the same time. It can be seen that the speed of the mixing paddle should not be too low, and the mixing speed should not be blindly increased to reduce the mixing time and improve production efficiency. This simulation conclusion is consistent with the actual mixing production of imitation marble, and the speed of the paddle is usually set within the process setting value of 40 rpm to 60 rpm. Therefore, it is feasible to study the influence of rotation speed on the mixing uniformity of materials by discrete element software simulation analysis method, which can provide a certain theoretical reference for the optimization of the imitation marble mixing production process.

### 4.3. Feasibility of Pattern Stirring Molding Simulation Experiment

**Figure 8:**
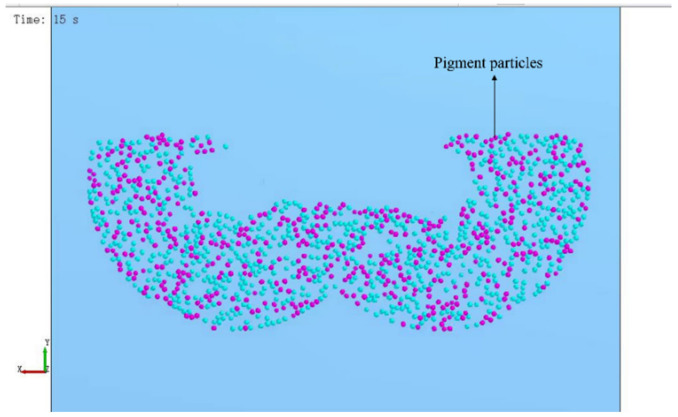
Particle distribution of substrate and patterned material.

In the experiment, the imitation marble pattern of “Aimeiyu” was used as the analysis object, and the simulation was carried out according to the same process as the actual production of “Aimeiyu” products. In the actual production, the stirring of the “Aimeiyu” pattern is to add the pigment to the stirred substrate, and then stir according to the process of stirring for 50 s, stopping for 60 s, stirring for 50 s, stopping for 60 s, stirring for 50 s, and stirring for 50 s to facilitate simulation experiments, and the stirring time is reduced by ten times. The particle distribution of the two materials after stirring is shown in Figure 8, where the blue particles represent the substrate and the purple particles represent the pigments.

**Figure 9:**
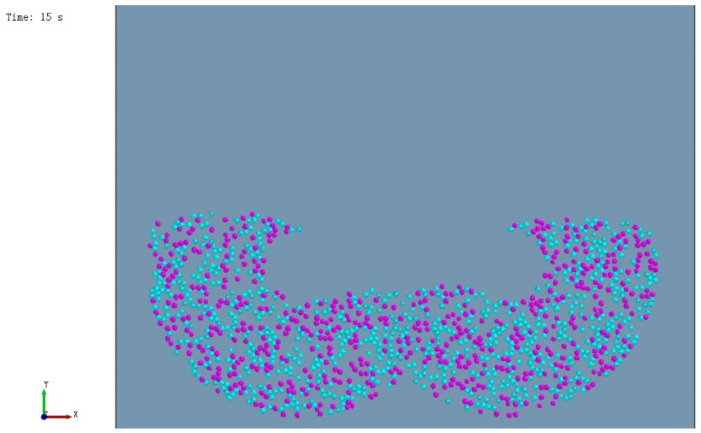
Particle distribution of the substrate and pattern material after the change.

Considering that the viscosity of unsaturated polyester resin will gradually increase and tend to become a stable viscosity state with the continuation of mixing after the addition of epoxy resin particles and a variety of cooperating agents, the recompiled cohesion model was added on the original basis to make the viscosity first grow linearly and then tend to be stabilized, and the results are shown in Figure 9.

Figure 9 shows an increase in the compactness of the pigment particles and a more textural effect compared to Figure 8, which indicates that the changed simulation experiment is more intuitive and consistent with the actual state compared to the previous one.

The actual pattern of “Aimeiyu“ imitation marble products is shown in Figure 9.

**Figure 10:**
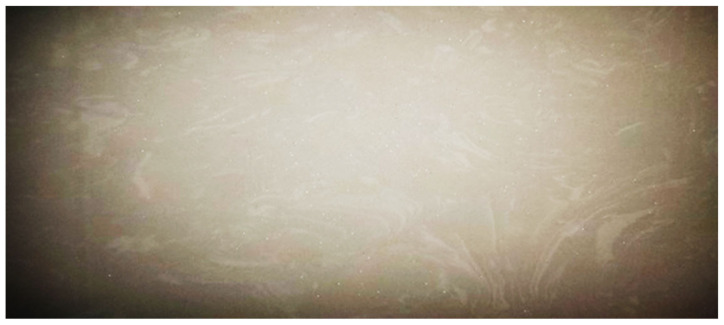
“Aimeiyu” imitation marble.

By comparing Figure 9 and Figure 10, it can be found that the trajectory of pigment particle distribution is similar to the trajectory of actual artificial marble patterns, indicating that simulating patterns using discrete element software EDEM is feasible.

## 5. Pattern Stirring and Molding Mechanism

### 5.1. Movement Trajectory of Pigment Particles

Trajectory analysis of particles plays an important role in guiding industrial production and equipment-related design and research [40,41].

**Figure 11:**
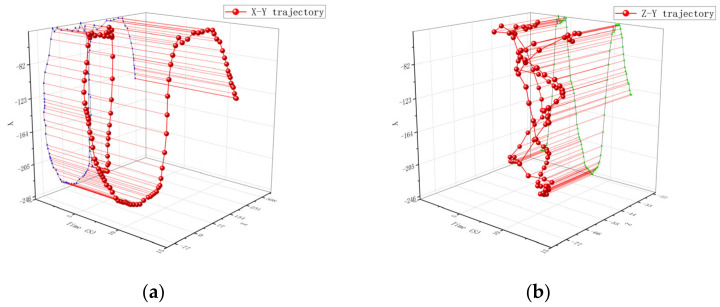
Single particle motion trajectory on the propeller head: (**a**) represents the trajectory changes and projections in the X-Y direction and (**b**) represents the trajectory changes and projections in the Z-Y direction. Where the blue line is the projection of the particle motion trajectory in the X-Y direction and the green line is the projection of the particle motion trajectory in the Z-Y direction.

**Figure 12:**
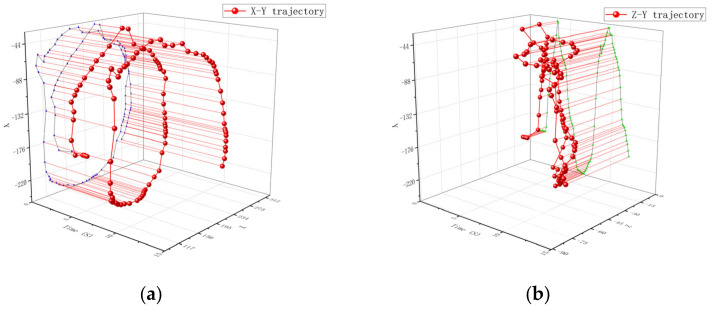
Single Particle Motion Trajectory on the Propeller Rod: (**a**) represents the trajectory changes and projections in the X-Y direction and (**b**) represents the trajectory changes and projections in the Z-Y direction. Where the blue line is the projection of the particle motion trajectory in the X-Y direction and the green line is the projection of the particle motion trajectory in the Z-Y direction.

**Figure 13:**
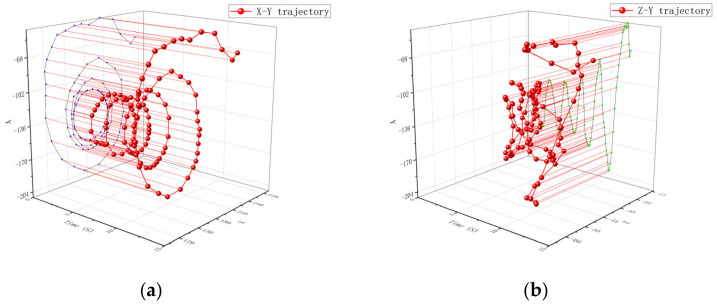
Single particle motion trajectory at the root of the propeller shaft: (**a**) represents the trajectory changes and projections in the X-Y direction and (**b**) represents the trajectory changes and projections in the Z-Y direction. Where the blue line is the projection of the particle motion trajectory in the X-Y direction and the green line is the projection of the particle motion trajectory in the Z-Y direction.

The essence of analyzing the formation of imitation marble patterns is to study the movement trajectory of pigment particles in the mixing chamber. Due to the different structures of the mixing paddles at different positions, the particle movement trajectories formed during stirring are different. Due to the large number of particles in the warehouse and the complex trajectory of multiple particles, it is difficult to distinguish the motion change trend, hence the selection of single particles in different positions for motion trajectory simulation is more helpful in analyzing the trajectory change trend, which provides a theoretical reference for pattern customization. The motion path of a single particle on the propeller head, rod, root, and stirring shaft was simulated for 12 s with the optimum speed range of 60 rpm. The results are shown in Figure 10, Figure 11, Figure 12 and Figure 13, where A represents the starting point and B represents the endpoint.

The movement track of a single particle at the propeller head is shown in Figure 11. From Figure 11a, it can be seen that the particles at the propeller head move in a circular motion in the radial direction. The movement track is relatively uniform, and its circular radius is similar to that of the agitator. Figure 11b shows that the particles have a larger amplitude of axial motion, which is caused by the inclination of the propeller head. Therefore, the axial trajectory of the particles will deviate significantly as they rotate with the propeller.

The movement track of a single particle at the propeller rod is shown in Figure 12. From Figure 12a, it can be seen that the movement of particles starts from the starting point. The track gradually tends to follow a circular motion similar to that at the propeller head, and the movement amplitude changes greatly. From Figure 12b, it can be concluded that the trajectory of particles in the axial direction changes relatively little. The motion trajectory of a single particle at the root of the propeller is shown in Figure 13. From Figure 13a, it can be concluded that the particles have a similar trend of change as that at the propeller shaft, and the particle trajectory in Figure 13b is also similar to that at the propeller shaft.

**Figure 14:**
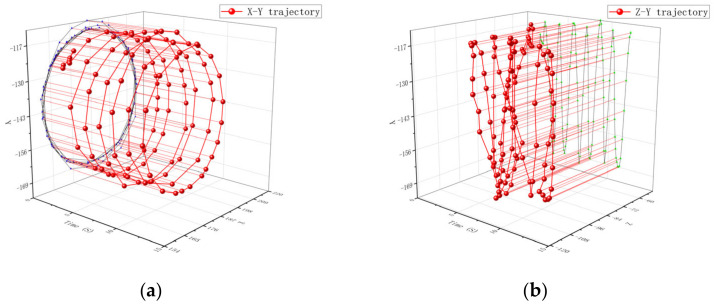
Single particle motion trajectory on the mixing shaft: (**a**) represents the trajectory changes and projections in the X-Y direction and (**b**) represents the trajectory changes and projections in the Z-Y direction. Where the blue line is the projection of the particle motion trajectory in the X-Y direction and the green line is the projection of the particle motion trajectory in the Z-Y direction.

The trend of the movement trajectory of a single particle on the stirring shaft is shown in Figure 14. From Figure 14a, it can be concluded that the particles have a similar movement trend in the radial direction as that at the paddle. From Figure 14b, it can be seen that the axial motion amplitude of particles on the shaft is relatively small because the particles on the stirring shaft are not affected by the stirring element and the forces are all from the viscous force exerted by the surrounding particles, which is prone to the phenomenon of excessive pigment in this area. This phenomenon is not conducive to the blending of patterns. Therefore, when customizing patterns, it is advisable to avoid adding pigments directly to the stirring shaft as much as possible.

### 5.2. Verification of Particle Motion Trajectory 

**Figure 15:**
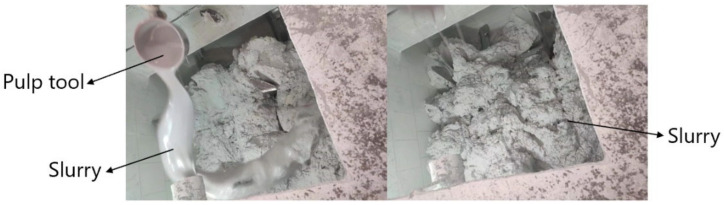
Blade trajectories formed after slurry and slurry splashing.

In this paper, the imitation marble pattern and its formation process of the “Yashi White” variety are used as the analysis object to demonstrate the reliability of the trajectory simulation experimental results. The formation of the “Ascotwhite” silt plate pattern is mainly achieved by two steps: pulping and stirring. The process is simple, and the results are easy to compare and analyze. Slurry throwing refers to the artificial splashing of simple, mixing pigments to the homogeneous substrate, and then mixing and stirring with the substrate. Since the color state of the slurry is different from that of the substrate, its movement trajectory becomes the pattern on the silt plate, as shown in Figure 15.

**Figure 16:**
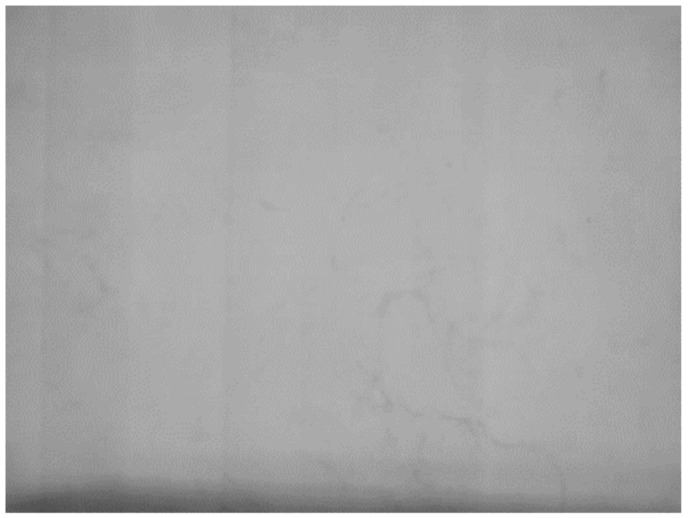
Finished pattern of the “Yashi White” imitation marble.

To facilitate spilling during the grouting process, most of the slurry is sprinkled on the base material between the two shafts, and part of it is sprinkled on the mixing shaft and the root of the paddle shaft. When stirring, the slurry sprinkled on the base material between the two shafts is mostly at the head of the oar and the paddle shaft, hence the splashing process can be regarded as the process of splashing the oar head and the paddle shaft. Then, vacuum stirring, pressing and forming, and cutting and polishing are performed to form a finished product, which is shown in Figure 16.

Most of the patterns formed after pouring are shallow and short, and some positions have darker colors and longer lines. Through the analysis of the pattern stirring and molding process, it can be seen that the reason for the formation of this situation is that when the pulp is splashed, there is more slurry at the position of the paddle and the paddle head, because the axial movement of the particles at this position is more violent, and the slurry is less than the substrate, hence the trajectory pattern distribution is less and shorter, and because the radial movement trend is similar, the radial motion is evenly distributed so that the pattern color is lighter. As for the slurry of the mixing shaft and root, due to the small amplitude of the particle trajectory movement, the distribution of the slurry is more concentrated after molding, so that the color of the formed pattern is darker.

By comparing with the actual imitation marble pattern, the particle trajectory analysis results and the pattern production process are mutually confirmed, and the analysis results explain the cause of the “Yashi White” granite pattern, and the granite pattern verifies the reliability of the analysis results, thus providing a theoretical reference for the imitation marble pattern customization.

## 6. Conclusions

In this paper, the mixing uniformity of particles is analyzed by the discrete element software, the feasibility of simulation experiments is verified, and the stirring and molding mechanism of particles is analyzed. The following results are obtained:

(1) With the increase in the speed of the mixing paddle, the mixing rate and mixing uniformity of the substrate will also increase. However, a speed too high will reduce the mixing efficiency, hence the speed of the mixing paddle should not be too low, let alone blindly increasing the mixing speed to reduce the mixing time and improve production efficiency. The optimal speed range of the mixing paddle is 40–60 rpm.
(2) The paddle head and the shaft part have a similar and regular trajectory change trend, which is of great significance for the study of imitation marble patterns.
(3) Combining the JKR model with the cohesion model is better than expressing the JKR model alone.
(4) Since the force of the particles on the mixing shaft comes from the surrounding particles, the magnitude and sustainability of the force are unstable, and these instabilities are not conducive to the study of customized patterns. Therefore, adding pigments directly to the mixing shaft should be avoided when conducting custom pattern research.

These conclusions can provide information exchange for the development of custom molding process technology for imitation marble products and provide theoretical references for the customized molding of artificial imitation marble.

Due to the large number and complex shape of powder particles in the actual production, the simulation experiment takes a long time, and it is difficult to reproduce the process in equal proportions. Therefore, the simulation experiment can only simplify the number of particles and particle shapes to represent the “particle clusters”, which leads to errors in the experiment. In the next research, firstly, the simulation process can be accelerated by optimizing the software; secondly, the shape of the particles can be recompiled to make the shape of the particles have complexity and versatility, and then by recompiling the API file of the contact model to make it more in line with the experimental environment at that time; Finally, the simulation process can be improved by combining the entire process of feeding, mixing, using various catalysts and compatibilizers, and adding pigments to more accurately simulate the particle clusters. The production process can be more accurately represented. Of course, the process depends more on the realization of the first step.

## Figures and Tables

**Table 1:** Various parameters of the mixing silo.

Type	Numerical Value
Poisson’s ratio	0.3
Shear modulus	77 Gpa
Density	7900 kg/m^3^
static friction coefficient	0.3
coefficient of rolling friction	0.01

**Table 2:** Particle parameters in simulation experiments.

Type	Numerical Value
Composite spherical particle size	5 mm
Density	2980 kg/m^3^
Shear modulus	2.5 × 10^10^ Pa
static friction coefficient	0.5
coefficient of rolling friction	0.01
Collision Coefficient of restitution between particles	0.5
Collision Coefficient of restitution with the vessel wall	0.5

**Table 3:** Materials represented by the two types of particles in the experiment.

Experiment Type	Triple-Composite Spherical Particles	Four-Composite Spherical Particles
Mixing uniformity analysis	powder	sand
Feasibility analysis of pattern simulation	pigment	Substrate
Single particle trajectory simulation at different locations	pigment	Substrate

## Data Availability

The raw/processed data required to reproduce these findings cannot be shared at this time due to legal or ethical reasons.
